# Multifaceted responses to two major parasites in the honey bee (*Apis mellifera*)

**DOI:** 10.1186/1472-6785-13-26

**Published:** 2013-07-17

**Authors:** Kaira M Wagoner, Humberto F Boncristiani, Olav Rueppell

**Affiliations:** 1 Department of Biology, University of North Carolina at Greensboro, Greensboro, USA

**Keywords:** Honey bee health, Host-parasite coevolution, Insect immunity

## Abstract

The recent declines in managed honey bee populations are of scientific, ecological and economic concern, and are partially attributed to honey bee parasites and related disease. McDonnell et al. investigate behavioral, chemical and neurogenomic effects of parasitization by the ectoparasite *Varroa destructor* and the endoparasite *Nosema ceranae*. The study reveals important links between underlying mechanisms of immunity and parasitization in social insects by demonstrating that chemical signals and neurogenomic states are significantly different between parasitized and non-parasitized honey bees, and that neurogenomic states are partially conserved between bees infected with distinct parasites. However the study does not reveal whether differences measured are primarily the result of adaptive host responses or of manipulation of the honey bee host by the parasites and/or confounding viral loads of parasitized individuals. Questions answered and raised by McDonnell et al. will lead to an improved understanding of honey bee health and, more generally, host-parasite interactions.

## Background

The honey bee (*Apis mellifera*) is important both as a model organism in scientific research [1] and as a pollinator of large-scale agricultural crops [2]. The recent declines in managed honey bee populations [3] have precipitated numerous studies on potential mechanisms. The problem of honey bee population decline appears to be heterogeneous and due to multiple, potentially interacting effects. The most likely contributors include general management stress, xenobiotic exposure, malnutrition, parasites and disease. Particular focus has been devoted to parasites and pathogens because honey bees lack many immune effector genes [1] and may be particularly susceptible to disease because they experience a high contact rate between closely related nestmates within colonies [4] and in modern apiculture also between colonies and populations.

Social organization results in an increased risk of disease transmission but it also adds a layer of defense, labeled social immunity [5]. Including physiological, behavioral, and organizational adaptations that reduce disease transmission within the colony, social immunity plays a critical role in honey bee health and might concomitantly explain fundamental aspects of honey bee social organization, such as the temporal division of labor among workers. In general, diseased individuals exhibit an earlier transition from in-hive tasks to foraging [6], which could be adaptive because it reduces disease transmission [4] and individuals with a lower residual life expectancy fulfill the risky task of foraging [7]. However, this response could also be due to parasite manipulation of its host to increase transmission [8] by entering uninfected hives due to homing errors. Little is known about the mechanisms of this accelerated maturation and its distinction from alternative hypotheses, such as altruistic self-removal [9] and eviction by nestmates [10].

A novel study [6] sheds light on the issue by investigating multiple aspects of honey bee workers that are parasitized by two of the most harmful honey bee parasites: *Varroa destructor* and *Nosema ceranae*. This comparison is particularly interesting because the former is a brood ectoparasite and the latter is an endoparasite of adult honey bees. McDonnell et al. find no changes in a series of behaviors, but report significant changes in the cuticular hydrocarbon profile and the brain transcriptome of infected workers. The transcriptome response in the brain of *Varroa* and *Nosema* infected workers is similar, suggesting a conserved neurological response to these two distinct parasites [6]. Alternatively, changes could be induced by the parasite-associated viruses.

In either case, McDonnell et al. reveal important links between underlying mechanisms of immunity and parasitization in social insects by demonstrating that chemical signals and neurogenomic states are significantly different between parasitized and non-parasitized honey bees. This is remarkable because neither parasite directly affects either brain or exocrine glands. Additionally, McDonnell et al. present evidence for upregulation of several immune-related genes in parasitized honey bees, suggesting that at least a portion of the physiological response is a defense against the parasites rather than a negative consequence of infection. The results suggest that the parasite-induced transcriptome changes are unrelated to the normal life-history transition from the in-hive to the foraging stage but the behavioral analysis finds no evidence for a forceful eviction of the parasitized individuals. The authors conclude that altruistic self-removal is most compatible with these results, but this conclusion must remain speculative in light of the small amount of neurogenomic overlap between experimental treatments that induce precocious foraging [11].

As a valuable contribution, the study by McDonnell et al. raises a number of further questions regarding the specific case of honey bee health and host-parasite interactions in general. Specific to the study, it would be interesting to know how comparable results from the natural hive and cage environment are, whether behavioral differences between treatment groups would have been picked up with a wider screen of the behavioral repertoire, and how changes in cuticular hydrocarbons relate between the bees infected with different parasites and a normal age-progression. Confounding variables such as different ages at parasitization and the presence of two major viruses in parasitized individuals also complicate the interpretations [12] and need to be subject of further study. Furthermore, the study cannot distinguish between adaptive host responses and manipulation of the honey bee host by the parasites, which is an urgent distinction given the practical importance of honey bee parasites and their potential as a model system. This study prompts the more general question how much can be learned about common disease and stress responses in parasitized hosts by comparing different biological aspects and disparate parasites. What experimental designs are required to yield truly integrated insights? Although these and other questions remain, this research marks significant progress in understanding the interactions between honey bees and their parasites, encouraging numerous future studies.

The study by McDonnell et al. is valuable to the increasingly important field of honey bee health. However, the findings also have implications for more general topics, such as the development and evolution of insect immune systems and life history. Findings like those of McDonnell et al. enhance our understanding of the connection between environmental stimuli and physiological adaptation, both in terms of immediate and predictive adaptive responses, in which early-life stimuli elicit anticipatory adaptations that confer fitness to similar stimuli experienced later in life [13]. A greater understanding of the commonalities and differences in immune response to different pathogen types and at different host ages may lead to improvements in understanding disease ecology and evolution and may be useful in both disease prevention and treatment.

## Competing interests

The authors declare that they have no competing interests.

## Authors’ contributions

KMW wrote the initial draft and all authors contributed to writing the final version of the manuscript and approved it.
